# Clinical presentation of bone tumours in children and young people: a systematic review and meta-analysis

**DOI:** 10.1136/archdischild-2024-327879

**Published:** 2025-02-05

**Authors:** Jo-Fen Liu, Dhurgshaarna Shanmugavadivel, Ashley Ball-Gamble, David Walker

**Affiliations:** 1 Children’s Cancer and Leukaemia Group, Leicester, UK; 2 Lifespan and Population Health, University of Nottingham, Nottingham, UK; 3 Children’s Brain Tumour Research Centre, University of Nottingham, Nottingham, UK

**Keywords:** Paediatrics, Child Health

## Abstract

**Background:**

Children and young people (CYP) with bone tumours often experience lengthy intervals prior to diagnosis that can lead to increased morbidity and mortality. Early diagnosis is key to optimising treatment options and long-term outcomes. This review aims to describe symptomatology at diagnosis, in order to develop interventions to accelerate diagnosis.

**Methods:**

A literature search of MEDLINE and EMBASE was conducted for studies published between January 2008 and May 2023, reporting signs and symptoms in CYP with bone tumours. Pooled proportions of symptoms and signs were calculated.

**Results:**

16 studies (1452 patients; 492 osteosarcoma and 932 Ewing’s sarcoma) were included in the analysis. The most prevalent symptoms were pain (64%, 95% CI 52% to 75%) and swelling (22%, 95% CI 6% to 42%). Other symptoms included fever, pain and swelling, history of trauma, pathological fracture, palpable mass, functional limitation, increased volume and limp. Subanalysis identified differences in symptom clusters: osteosarcoma more frequently presented with a history of trauma, pathological fracture, pain on weight-bearing, pain worse at night, pain at rest and weight loss. Ewing’s sarcoma was associated with fever, functional limitation and a palpable mass.

**Conclusions:**

These data highlight the differences in presentations between osteosarcoma and Ewing sarcoma, which may account for differences in survival and outcome. This will be used to inform professional and public health interventions through the Child Cancer Smart campaign to accelerate diagnosis for all. This review also highlights the need for a renewed research focus to identify patients earlier in the disease development as a strategy to improve the current static outcomes.

**WHAT IS ALREADY KNOWN ON THIS TOPIC**

- Bone tumours in children and young people (CYP) have lengthy diagnostic intervals which will affect staging at diagnosis, treatment intensity and long-term morbidity and mortality.
- Survival rates have not improved in the last 15 years, despite advancements in treatment modalities.
- Awareness of symptom recognition has been proven through the HeadSmart campaign to reduce prediagnostic interval and provides the best possible outcomes for these CYP.

**WHAT THIS STUDY ADDS**

- This review provides an evidence base for presentation of CYP with bone tumours and the differences between Ewing and osteosarcoma. It also identifies a paucity of studies focusing on this topic in the last decade.

**HOW THIS STUDY MIGHT AFFECT RESEARCH, PRACTICE OR POLICY**

- The findings will guide the development of clinical guidelines to aid early recognition and diagnosis of bone tumours in CYP, improving referral processes and patient outcomes through timely intervention.

## Introduction

 About 1 in 450 children will develop cancer by the age of 15, and by the age of 20, this cumulative risk increases to 1 in 350.^1^ Bone tumours account for approximately 3%–4% of all childhood cancers,^2^ osteosarcoma and Ewing sarcoma are the most common subtypes, representing 52% and 34% of the cases, respectively. This equates to an average of 69 cases per year for children aged 0–14 years and 88 cases for those aged 15–24 years in the UK.^2^ Incidence of osteosarcoma increases with age, peaking during puberty, while Ewing’s sarcoma occurs throughout childhood and can additionally arise in soft tissues. Geographical differences in incidence and distribution of bone tumour subtypes have also been reported,^3^ highlighting the potential complex interplay of genetic, environmental and/or sociodemographic factors in disease development.

The introduction of multidisciplinary strategies combining limb preserving surgery, widespread testing and use of chemotherapy in international trials and selected use of radiotherapy, has led to a rise in survival rates which has plateaued in the past decade.^24^,^6^ Despite these early advances, bone tumours as a group have the lowest survival rates among the major childhood cancer types, with a 5-year survival of 69% for osteosarcoma and 53% for Ewing’s sarcoma in patients aged 0–24 year^2^. Furthermore, there has been little significant progress in 10-year survival rates for children with metastatic disease between the 1990s and 2000s (osteosarcoma at 23.9% vs 29.3% and Ewing’s sarcoma at 30.6% vs 32.2%).^4 7^ This lack of progress for osteosarcoma and Ewing sarcoma has also been noted in the more recent EUROcare-6 data, covering children and young people (CYP) with cancer in Europe up to 2014.^8^

A second factor that is persistently reported across health systems is the prolonged prediagnostic intervals associated with clinical presentation of bone tumours. A systematic review reported a pooled median time to diagnosis longer for Ewing’s sarcoma (14.1 weeks) than for osteosarcoma (9.1 weeks); and clinician-related interval accounting for 67% of the total diagnostic time in Ewing’s sarcoma compared with 34% for osteosarcoma.^9^ This is in comparison to a pooled median time of 2.7 weeks for leukaemia, 3.3 weeks for neuroblastoma, 6.4 weeks for rhabdomyosarcoma and 13 weeks for soft tissue sarcomas.^9^ The BRIGHTLIGHT study of teenage cancer presentations found that 25% of young people with bone tumours waited over 1 month, and 9% waited over 3 months before consulting a healthcare professional.^10^

Cancer is often overlooked as a possible diagnosis in CYP. In high-income countries, children make up a minority of the population seen by primary care physicians diluting awareness and clinical experience of paediatric priorities. Musculoskeletal symptoms are infrequent in young people and symptoms related to inflammation, growth and injury need to be discriminated from the persistent and progressive symptoms of bone malignancy. As part of a UK national strategy to enhance cancer symptom awareness in CYP, the Children’s Cancer and Leukaemia Group (CCLG) referral guidance for CYP with suspected cancer has been published as an addendum to The National Institute for Health and Care Excellence (NICE) guidance after calls for greater clarity for CYP.^11^ We have performed a systematic review of symptomatology of childhood bone tumour presentation to inform a planned public and professional awareness initiative called Child Cancer Smart where tumour-specific symptom awareness and prediagnostic intervals will be used as a marker of the programme’s impact.

## Methods

This review was conducted in alignment with Preferred Reporting Items for Systematic reviews^12^ and Meta-Analysis and Strengthening the Reporting of Observational Studies in Epidemiology guidance.^13^

### Review question

What are the signs and symptoms of bone tumours in CYP under the age of 18?

### Search strategy

MEDLINE and EMBASE were searched without language restriction, from January 2008 to May 2023. Keywords were: ‘bone tumour(s)’, ‘bone tumor(s)’, ‘bone neoplasm(s)’; ‘Ewing(s)’; ‘osteosarcoma’ and ‘diagnosis’; ‘presentation’ and ‘sign(s)’ or ‘symptom(s)’. Retrieved references were restricted to ‘human’ and age (‘infants’, ‘newborns’, ‘infant or child’, ‘preschool or child’ and ‘adolescent’). For full search terms and strategy, see online supplemental table S1.

### Selection criteria

Cross-sectional studies, case series or cancer registry reports, with information about tumour presentation, tumour diagnosis or clinical signs/symptoms in patients aged 0–18 years and published after January 2008 were included. Conference abstracts were included only if sufficient information was available from the abstract alone.

Exclusion criteria were: case reports, letters to the editor, editorials or articles without abstract; studies combined adult and paediatric data or no paediatric data; studies with less than 10 patients; studies lacking sufficient information or primary data; studies on unrelated subjects; and full text not available through interlibrary loan service. A comprehensive approach to identify grey literature was adopted, including searching reference lists and contacting authors.

### Literature screening

Eligibility screening and literature review was carried out by an independent researcher (DS) and agreed with another researcher (J-FL).

### Data extraction

A standard pro forma was set up to record data on year of publication, country, recruitment period, number of patients, study design, data source, tumour location, age and symptom presentation (as described in the individual study). Non-specific and similar symptom clusters were grouped together. Symptoms reported as combined categories or with detailed information (eg, ‘pain and swelling’) that could not be reclassified were included in the meta-analysis as reported. If a sign/symptom was not recorded in a study, it was assumed not to occur in that population. All data were extracted by a researcher (DS) and quality-checked by another researcher (J-FL) and a senior paediatric oncologist (DW).

### Quality assessment

The quality of eligible studies was assessed by evaluating the following domains: recruitment period, number of institutions, sample selection, case definition, data ascertainment and quality of reporting (online supplemental table S2). The form is a combined modification of crucial methodological domains used in pre-existing quality assessment tools for observational studies, as described in our previous publication.^14^

### Statistical analysis

Pooled proportions (%) of children presenting with each sign or symptom at diagnosis were estimated using MetaXL V.5.3 (EpiGear International, Queensland, Australia). Given the high heterogeneity across the eligible studies included in this review, the analysis employed the random effects model (DerSimonian-Laird method) and Freeman-Tukey double arcsine transformation to calculate pooled proportions. Heterogeneity was assessed using the *I*² statistic.

A predetermined threshold was set for symptoms/signs reported in 2% or more of the cohort. This helps to compromise between identifying clinically relevant symptoms and minimising the potential risk of overinterpreting non-specific symptoms. Analysis of all bone tumours was undertaken to provide a summary of tumour presentation. Subanalysis by tumour type and age group was also carried out when feasible.

## Results

**Figure 1:**
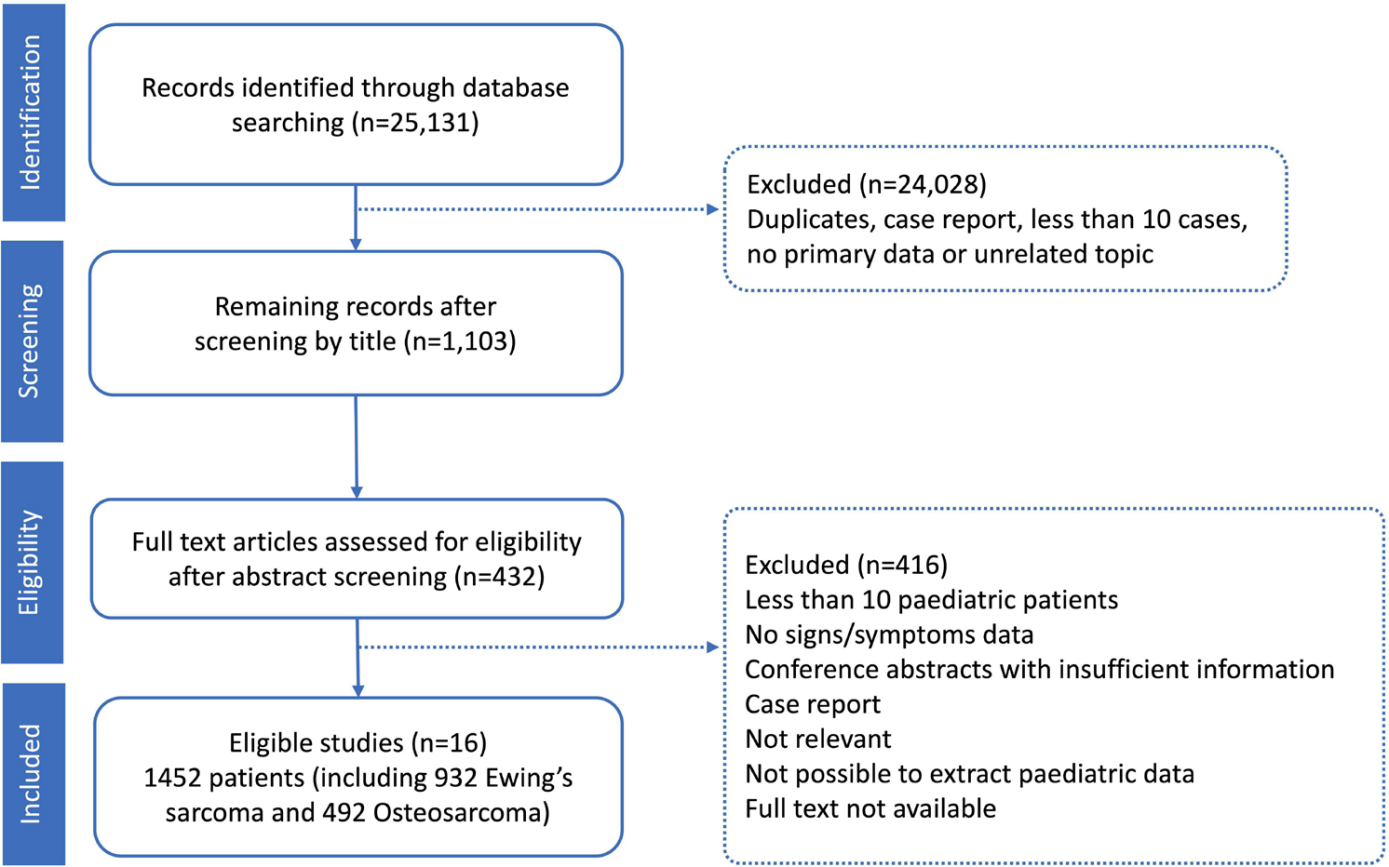
Progress through the meta-analysis.

The literature screening process is shown in figure 1. The initial search identified 25 131 publications. After title and abstract screening, 432 were eligible for full-text review. Another 416 articles were further excluded after full-text screening based on predefined criteria, leaving a total of 16 studies included in the final analysis.^15^,^30^ These studies provided data on 1452 patients, including 932 with Ewing’s sarcoma and 492 with osteosarcoma, across 12 countries (online supplemental table S3). Eight studies had a small number of young people older than 18 years of age. Given that bone tumours are more prevalent in adolescents and young adults than children, we decided to include these studies in the analysis.^1520^,^23 25 27 30^

The quality was comprehensively evaluated and summarised (online supplemental table S4). A total of 38 symptoms/signs were recorded. Symptoms/signs were recorded as prediagnostic (initial or at presentation) or at diagnosis in 13 studies; 3 studies did not specify when the symptoms/signs were identified.

### All cases

**Figure 2:**
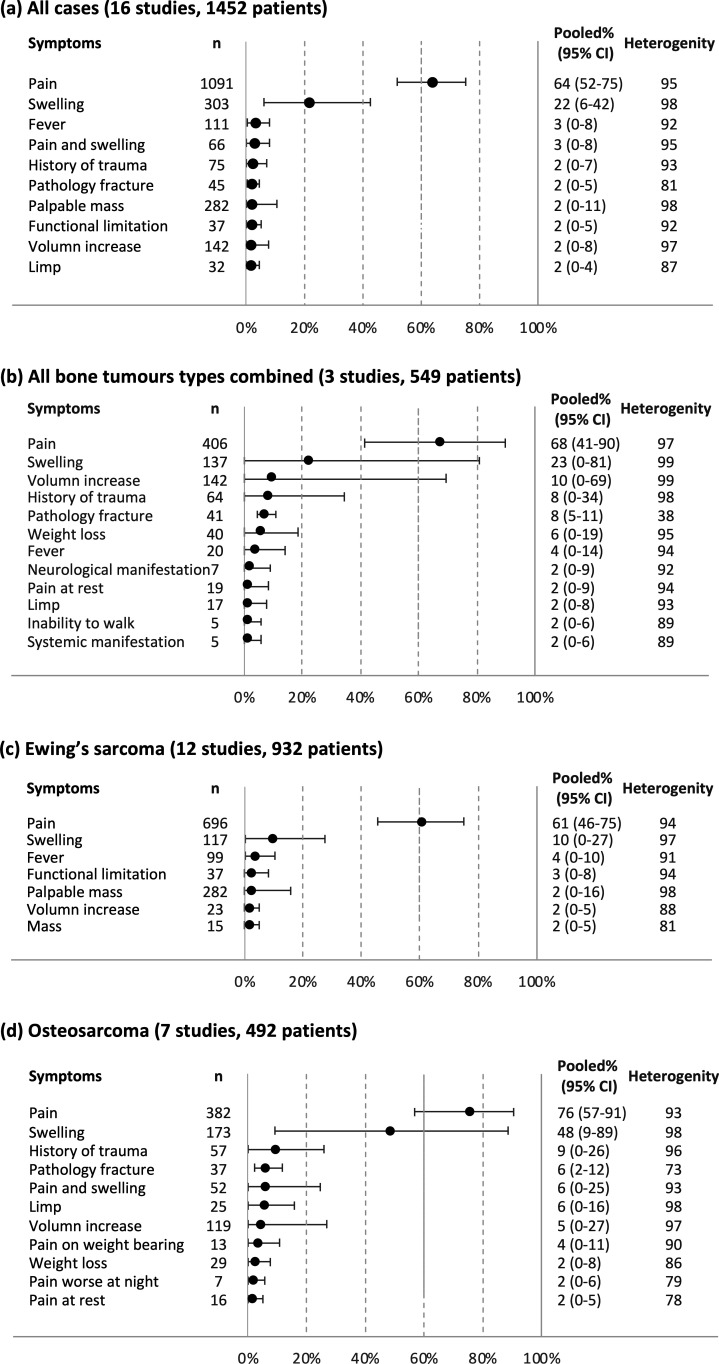
Pooled proportions for the most common prediagnostic signs and symptoms for bone tumour. (a) all cases from 16 eligible studies, (b) studies reported all bone tumour types, (c) Ewing’s sarcoma and (d) Osteosarcoma.

Figure 2a illustrates the pooled proportions of signs and symptoms in 1452 bone tumour patients across all 16 eligible studies.^15^,^30^ A total of 36 symptoms were recorded, of which 20 reached the predetermined 2% cut-off (for full results, see online supplemental table S5). The most common signs/symptoms were pain (64%) and swelling (22%). Other symptoms included fever (3%), pain and swelling (3%), history of trauma (2%), pathological fracture (2%), palpable mass (2%), functional limitation (2%), volume increase (2%) and limp (2%). All analyses showed high heterogeneity (*I*^2^ 81%–98%).

**Figure 3:**
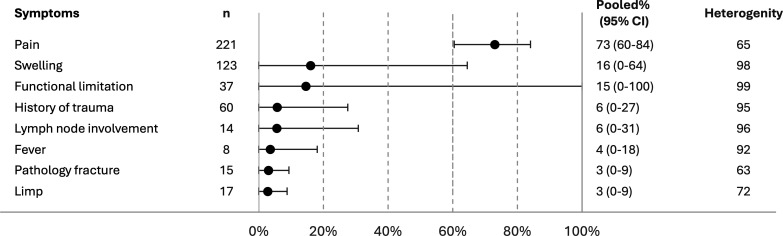
Pooled proportions for the most common prediagnostic signs and symptoms for bone tumour in children aged 0–15 years.

### All bone tumour types combined

Three papers reported signs and symptoms of all bone tumour types (n=549), including two single centre studies^15 26^ and one by regional children’s cancer registry data in the UK.^18^ The results were similar (figure 2b), with pain (68%) and swelling (23%) being the most common symptoms, followed by volume increase (10%), history of trauma (8%), pathology fracture (8%) and weight loss (6%). Other symptoms included fever (4%), neurological manifestation (2%), pain at rest (2%), limp (2%), inability to walk (2%) and systemic manifestation (2%).

### Ewing’s sarcoma

12 studies (n=932) reported signs and symptoms of Ewing’s sarcoma.^1518 20 21 23^,^30^ The most common symptoms were pain (61%) and swelling (10%). Other symptoms included fever (4%), palpable mass (2%), volume increase (2%) and mass (2%). Heterogeneity (*I*²) among studies ranged from 81% to 98% (figure 2c).

### Osteosarcoma

Figure 2d shows the pooled proportions of signs and symptoms in 492 paediatric osteosarcoma patients across seven studies.^15^,^1922 26^ The most common signs/symptoms were pain (74%) and swelling (48%), followed by history of trauma (9%), pathological fracture (6%), limp (6%) and volume increase (5%). Other symptoms included pain and swelling (4%), pain on weight-bearing (3%), weight loss (2%), pain worse at night (2%), and pain at rest (2%). Heterogeneity (*I*²) ranged from 73% to 98%.

### Age group

Subanalysis by age group was not feasible, as only one paper reported symptoms for children aged under 5.^19^ This was a multicentre study including 15 children under the age of five diagnosed and treated for bone tumours at seven cancer treatment member centres in France. The primary symptoms were pain (12/15, 80%), swelling (2/15, 13%) and fracture (1/15, 7%).

Signs and symptoms for children aged 0–15 years were also explored, since parents typically act as proxies, observing health changes and arranging medical appointments on behalf of their children. Three studies (n=325) were included in the analysis (figure 3).^18 19 24^ Pain (73%), swelling (16%), functional limitation (15%) were the most common, followed by history of trauma (6%), lymph node involvement (6%), fever (4%), fracture (3%) and limp (3%).

### Income group

**Figure 4:**
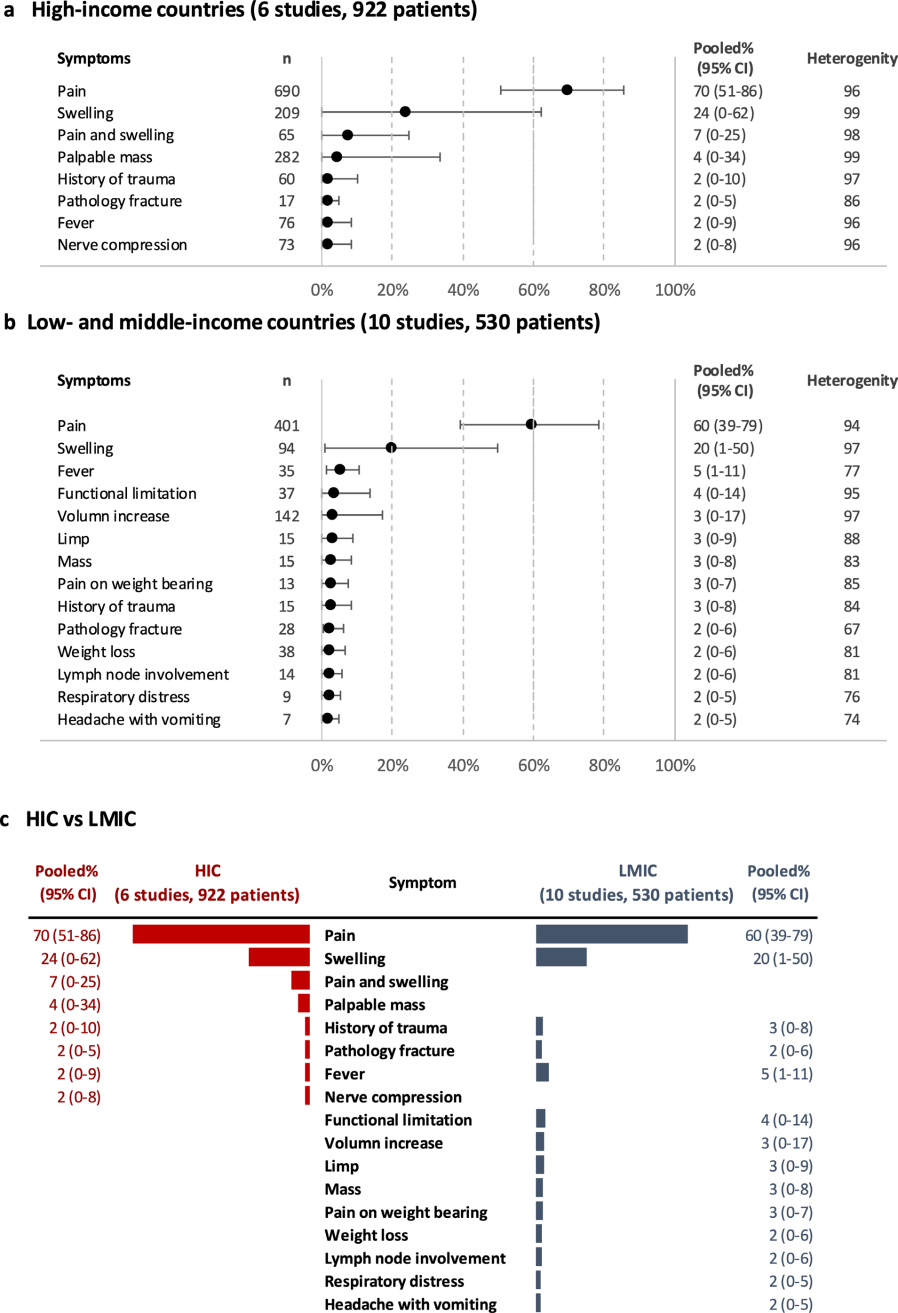
Pooled proportions for the most common prediagnostic signs and symptoms for bone tumour. (a) high-income countries (HICs), (b) low-income and middle-income countries (LMICs) and (c) comparison between HIC and LMIC.

Signs and symptoms reported in low-income and middle-income countries (LMICs) were more diverse than that in high-income countries (HICs) (35 vs 14). Of these, 14 and 8 signs/symptoms had pooled proportions>2%, respectively (figure 4). Pain and swelling are the two most common symptoms in both HICs (70% and 24%) and LMICs (60% and 20%). Other features presented in both settings, with pooled proportions ranging between 2% and 5%, were history of trauma, pathology fracture and fever. All analyses showed very high heterogeneity (*I*² = 74%–99%). We cannot rule out the possibility that the differences observed are due to other confounders rather than the selected subgroup.

## Discussion

This review reports presentation symptoms of a large publication cohort of 1452 CYP with bone tumours diagnosed from 1957 to 2016. The overall results reflect previous literature and clinical experience, that bone pain and swelling are the most common presentation overall and also identifies differences in presentation of other symptoms between osteosarcoma and Ewing’s tumour.

Previous published data has highlighted lengthy intervals to diagnosis for bone tumours, with marked differences between tumour types, which needs urgent focus to improve outcomes given that survival has remained static and behind other tumour types. This review provides a strong evidence base to use in developing clinical guidance and enhancing awareness in order to improve recognition of suspected bone tumours, accelerate diagnosis and improve outcomes for these CYP.

### Tumour type

**Figure 5:**
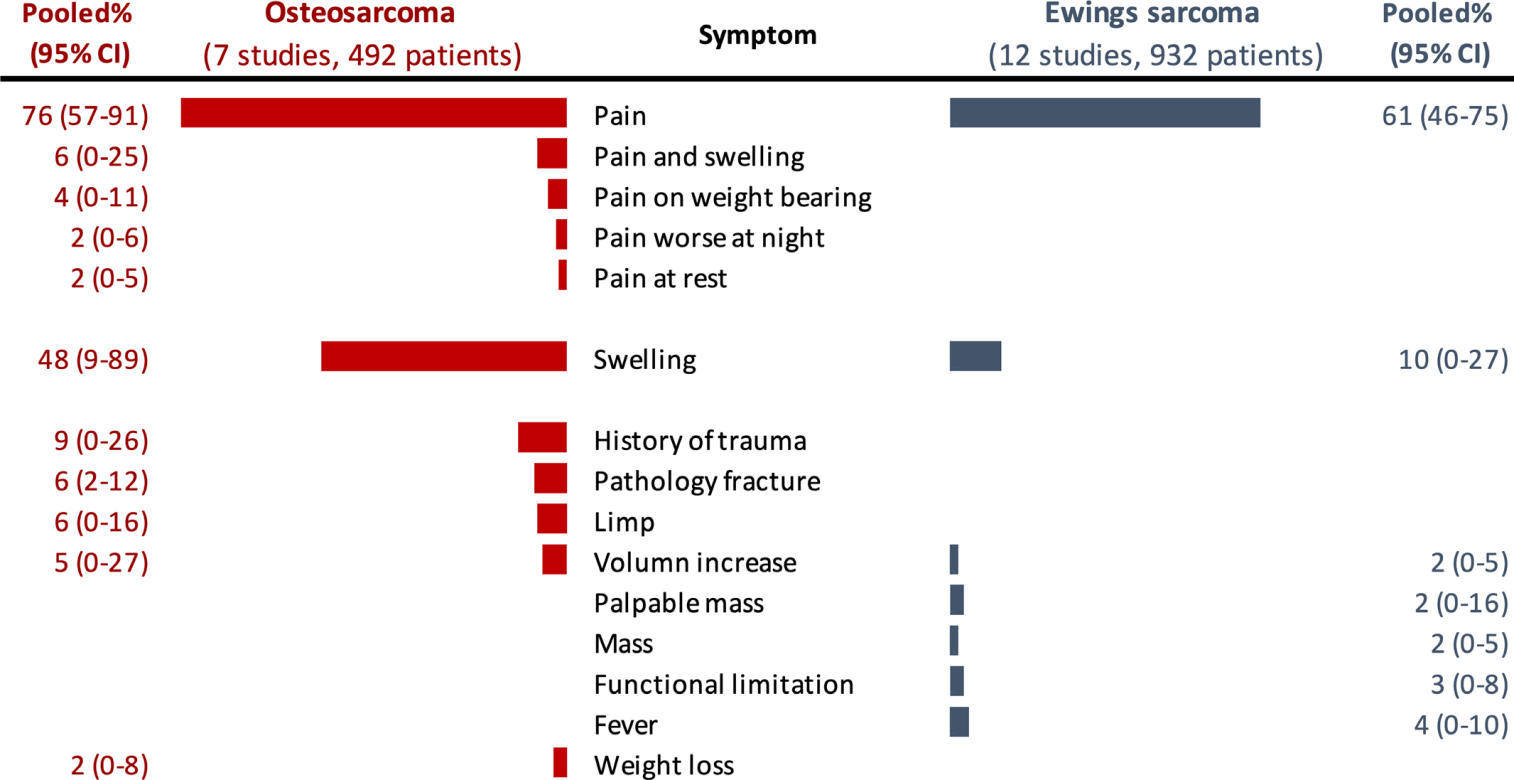
Comparison of pooled proportions of common symptoms between osteosarcoma and Ewing's sarcoma.

The subanalysis by tumour type highlights that aside from pain and swelling, the other symptomatology of osteosarcoma and Ewing sarcoma are distinct and could be the focus of content for public and professional awareness (figure 5). Osteosarcoma is associated with painful swelling and often presents with a limp or incidental fracture. There is also often a misleading history of trauma. In contrast, Ewing’s sarcoma more commonly presents with fever, poorly localised pain and soft tissue masses.

It is well recognised that osteosarcoma and Ewing’s sarcoma have different skeletal site distributions, which accounts for these differences in presentation. Osteosarcoma primarily affects the lower limb long bones and Ewing sarcoma presents in the axial skeleton including the pelvis, and ribs, but can also present in upper and lower limbs.^31^ Diagnosis of both tumour types rely on awareness of the importance of persistent/progressive musculoskeletal symptoms of pain, which justify imaging. Given that patients frequently present to general practice or the emergency department, understanding and educating on the breadth of non-specific symptoms with which bone tumours present, as well as the possibility of axial skeleton involvement will raise clinical suspicion, especially for those with repeated attendances, and facilitate earlier referral for diagnosis.

### Age group

The variation of age-specific, site-specific and tumour-specific incidence indicates a link to bone growth and physiological development of CYP. Unfortunately, subanalyses by age group were not feasible in this review due to the scarcity of studies focusing on bone tumours in younger children. We are acutely aware of the differences in presentations at different ages and acknowledge the contribution of the BRIGHTLIGHT study in identifying the challenges faced by young people with cancer accessing healthcare systems.

From a different perspective, help-seeking behaviour differs between children 0–15 years who mainly rely on parents as proxies, and those aged 16 and above, who seek medical advice independently. Although direct comparison was not feasible in this study, we reported symptom presentation in children aged 0–15 to help inform future awareness campaign and support parents in recognising symptoms. Future research should aim to further explore these differences and bridge the gap with more comprehensive data.

### Income group

Clinical presentation of bone tumour, aside from pain and swelling, differed between LMIC and HIC. Functional limitation, volume increase and limp were more common in LMIC suggesting greater disease bulk. This could be attributed to disease status and possibly to be associated with patient factors such as socioeconomic status and low symptom awareness or system factors such as delay in diagnosis and misdiagnosis. This needs further exploration in line with the WHO Global Initiative for Childhood Cancer.^32^,^35^

### Strengths and limitations

This systematic review is the first to describe the presentation of bone tumours in CYP. By employing an extensive search strategy without language restrictions, the study compiled data from over 1400 children in 12 countries with diverse income levels and clinical contexts, offering the most current and comprehensive insight into clinical features at presentation.

Cohort heterogeneity and the associated broad confidence intervals are a weakness of this study. The selected articles were all observational, with data derived primarily from hospital records. The data collection methods, including the timing (whether at symptom onset, presentation or soon after diagnosis) and how symptoms were described and recorded, were not standardised, raising the risk that less common symptoms might be underreported. In the analysis the non-reporting of a symptom was assumed to be absent, representing a risk of underestimation.

Furthermore, there was limited additional information such as detailed tumour locations and genetic predisposition states which would be helpful in assessing risk for individual patients. The absence of studies from North American is also noted, and the reason for this is unclear. While the clinical presentation characteristics are likely to be similar to those in other developed countries, differences in healthcare systems, referral and diagnostic practices, and population characteristics cannot be assumed to be negligible in their influence if interventions were to be introduced in future studies. When interpreting the results, it is important to bear these uncertainties in mind and view the findings as patterns rather than making direct rank comparisons or definitive conclusions to avoid over-interpretation.

### Implications of findings

The study, despite some limitations, provided evidence on the clinical presentation of bone tumours in CYP. We have been able to clearly describe the different symptom clusters associated with different tumour types. This information will help shape future tumour-specific clinical guidelines, supporting primary and secondary care healthcare professionals to be guided to select children for investigation of the possibility of bone tumours.^36^

The findings will also be used to support a national awareness campaign called Child Cancer Smart.^37^ Inspired by the successful HeadSmart campaign,^38^ this initiative aims to improve symptom recognition, reduce delays in accessing healthcare, and promote evidence-based guidelines for investigating suspected bone tumours in children, ultimately leading to better patient outcomes through timely intervention.

### Future research

This review is part of a drive for early diagnosis in childhood cancer. Delay in diagnosis is often a focus of complaints and legal processes, and it is well known that CYP with bone tumours experience prolonged prediagnostic intervals compared with other tumour types.^9 39^ However, our search highlights the paucity of available data for presentation of bone tumours with just 16 studies included in our review.

During the search, we also noted a lack of contemporary research on prediagnostic intervals. And so, there is a need for renewed focus on early diagnosis research to bridge the gap between prolonged time to diagnosis and outcomes of bone tumours, given the lack of improvement in survival for this subgroup. There are also research gaps in screening for predisposition states, targeted surveillance, and enhanced clinical awareness in childhood cancers. For instance, the prevalence of osteosarcoma during adolescent bone growth and genetic mutations in Ewing’s Sarcoma could provide clinical and biological markers for earlier detection. These efforts are essential for reducing prediagnostic intervals and bettering patient outcomes.

## Conclusions

This review provides an evidence base of the common presentations of bone tumours in CYP, highlighting differences between tumour types. These data will be used to develop clinical guidance and raise awareness through a new national awareness campaign called Child Cancer Smart in order to improve outcomes for bone tumours in CYP. Further research to understand the impact of intervals on outcomes is urgently required to close the gap for these CYP.

## Supplementary material

10.1136/archdischild-2024-327879 online supplemental file 1

10.1136/archdischild-2024-327879 online supplemental file 2

10.1136/archdischild-2024-327879 online supplemental file 3

10.1136/archdischild-2024-327879 online supplemental file 4

10.1136/archdischild-2024-327879 online supplemental file 5

## Data Availability

Data are available on reasonable request.
