# Supplementary material for: Clinical presentation of bone tumours in children and young people: a systematic review and meta-analysis
Source: Arch Dis Child. 2025 Feb 5;110(8):e327879. doi: 10.1136/archdischild-2024-327879 (PMC12320600; doi:10.1136/archdischild-2024-327879)
Supplement: online supplemental file 1 [file archdischild-110-8-s001.pdf]

Table S1 Literature review search terms and strategy

1. bone tumour*.ti,ab.
2. bone tumor*.ti,ab.
3. bone neoplasm*.ti,ab.
4. Ewing*.ti,ab.
5. osteosarcoma*.ti,ab.
6. exp Bone Neoplasms/
7. bone neoplasms/di
8. Sarcoma, Ewing/di
9. Osteosarcoma/di
10. or/1-9
11. diagnosis.ti,ab.
12. exp Diagnosis/
13. sign*.ti,ab.
14. symptom*.ti,ab.
15. exp "Signs and Symptoms"/
16. (signs and symptoms).ti,ab.
17. presentation*.ti,ab.
18. diagnos*.ti,ab.
19. or/11-18
20. 10 and 19
21. limit 20 to humans
22. limit 21 to ("all infant (birth to 23 months)" or "all child (0 to 18 years)" or "newborn infant (birth to 1 month)" or "infant (1 to 23 months)" or "preschool child (2 to 5 years)" or "child (6 to 12 years)" or "adolescent (13 to 18 years)")
23. limit 22 to (infant <to one year> or child <unspecified age> or preschool child <1 to 6 years> or school child <7 to 12 years> or adolescent <13 to 17 years>)
24. limit 23 to yr="2008 -Current"
25. remove duplicates from 24
