# Supplementary material for: Clinical presentation of bone tumours in children and young people: a systematic review and meta-analysis
Source: Arch Dis Child. 2025 Feb 5;110(8):e327879. doi: 10.1136/archdischild-2024-327879 (PMC12320600; doi:10.1136/archdischild-2024-327879)
Supplement: online supplemental file 2 [file archdischild-110-8-s002.pdf]

Table S2 Quality assessment tool - combined modification of crucial methodological domains used in pre-existing quality assessment tools for observation studies Critical Appraisal Skills Programme (CASP), Joanna Briggs Institute (JBI) critical appraisal tool, and Newcastle Ottawa Scale (NOS)

Methodological domains		Specific questions
**Recruitment period**		Not applicable
**Number of institutions**		Not applicable
**Sample selection**	Study populations	Was study population (inclusion/exclusion) clearly specified?<br>Were the reasons for exclusion reported and consistently applied?
	Sample strategy	Selection process – convenience sample, consecutive etc
**Case definition**	Standard definition of diagnosis	Were specified diagnosis criteria consistently applied?
	Verification of diagnosis	Did all patients receive biopsy to verified diagnosis or was it a clinical diagnosis?
**Ascertainment of data**	Source of data	Not applicable
	Management of missing data	Not applicable
**Quality of reporting**	Report type	Full text or conference abstract
	Level of symptom detail reported	Number of symptoms reported.<br>Were symptoms pre-specified?<br>How was it decided which symptom to report?
