# Supplementary material for: Clinical presentation of bone tumours in children and young people: a systematic review and meta-analysis
Source: Arch Dis Child. 2025 Feb 5;110(8):e327879. doi: 10.1136/archdischild-2024-327879 (PMC12320600; doi:10.1136/archdischild-2024-327879)
Supplement: online supplemental file 3 [file archdischild-110-8-s003.pdf]

Table S3 Characteristics of the 16 studies included in the analysis

Year of publication	Authors	Country	Income group[§]	N	Recruitment period	Design	Sources of data[*]	Number of institutions[$]	Tumour Type	Tumour location	Mean age (yrs)	Median age (yrs)	Age range (yrs)
2006	Guerra et al[15]	Brazil	UM	253	1985-2001	O	MR (S/T)	1	All	All	Osteosarcoma 15.7 Ewing's 12.8	-	0-30
2010	Ajura et al[16]	Malaysia	UM	12	1967-2008	O	MR (S/T)	1	Osteosarcoma	Jaw	-	-	6-18
2010	Pan et al[17]	Malaysia	UM	22	2003-2008	O	Interview (S/T)	1	Osteosarcoma	Knee	-	-	9-18
2010	Parkes et al[18]	UK	H	259	1957-2006	R	CR	West Midlands Regional Children's Tumour Registry	All	All	-	-	0-14
2011	Guillon et al[19]	France	H	15	1980-2007	O	MR (S/T)	7 SFCE centres[¥]	Osteosarcoma	All	-	3.9	1-4.9
2014	Akhavan et al[20]	Iran	LM	18	2002-2010	O	MR (NS)	1	Ewing's/ PNET	All	13.72	15	0-19
2014	Brasme et al[21]	France	H	436	1988-2000	T	Prospective analysis of two multi-centre trials	>2	Ewing's/ PNET	All	-	12 (IQR 9-15)	0-21
2015	Berlanga et al[22]	Spain	H	77	1985-2011	O	MR (S/T)	1	High grade Osteosarcoma	All	-	12.9	1.9-21.3
2015	Misra et al[23]	India	LM	21	1997-2014	O	MR (S/T)	1	Ewing's	Chest wall	9.9	-	3-20
2016	Caloretti et al[24]	Peru	UM	51	2006-2016	O	NS (S/T)	1	Ewing's	All	9	-	<15
2017	Nazeer et al. [25]	Egypt	LM	74	2004-2014	O	MR (S/T)	2	Ewing's	All	-	13	4-22
2018	Galila et al[26]	Egypt	LM	37	2003-2016	O	MR and Interview (S/T)	1	All	All	-	11 (IQR 5-14)	2-18
2018	Geiszl et al[27]	Hungary	H	78	2001-2013	O	MR (S/T)	1	Ewing's	All	11.6	11.97	0-20
2018	Chen et al[28]	China	UM	11	2003-2017	O	MR (S/T)	1	Ewing's/ PNET	Intracranial	-	-	1-18
2019	Majeed et al[29]	Iraq	UM	31	2009-2015	O	MR(NS)	1	Ewing's	All	-	13	2-18
2021	Liveralla et al[30]	Italy	H	57	1990-2020	R	MR (S/T)	1	Ewing's	Extraosseous	-	14	2-20

[§] Income group based on the World Bank Country and Lending Groups (https://datahelpdesk.worldbank.org/knowledgebase/articles/906519-world-bank-country-and-lending-groups)

*O: observational study;. R:cancer registry T: analysis of prospective trials

[$] MR: medical records; CR: cancer registry data; P: primary care data; S/T: secondary /tertiary care records; NS: not specified

[¥] Société Française des Cancers et leucémies de l'Enfant, (https://sf-cancers-enfant.com/)
