# Supplementary material for: Clinical presentation of bone tumours in children and young people: a systematic review and meta-analysis
Source: Arch Dis Child. 2025 Feb 5;110(8):e327879. doi: 10.1136/archdischild-2024-327879 (PMC12320600; doi:10.1136/archdischild-2024-327879)
Supplement: online supplemental file 4 [file archdischild-110-8-s004.pdf]

Table S4 Comprehensive findings from quality evaluation

Authors	Study population	Sample strategy	Case definition and verification	Ascertainment of data	Type of report	Level of symptom detail reported
Guerra et al[13]	Osteosarcoma and Ewing's<br><br>Inclusion/exclusion criteria: Yes	All consecutive patients during specified study period	Yes.<br><br>Histologically confirmed	Medical record (secondary/ tertiary)<br><br>Pre-diagnostic / clinical presentation at diagnosis	Full text article	Symptoms list (6)
Ajura et al[14]	Osteosarcoma<br><br>Inclusion/exclusion criteria: Yes	All consecutive patients during specified study period.	Yes.<br><br>Histologically confirmed	Medical record (secondary/ tertiary)<br><br>Pre-diagnostic/ clinical presentation at diagnosis	Full text article	Symptoms list (5)
Pan et al [15]	Patients with osteosarcoma around the knee joint (aged 9-34 yrs)	All consecutive patients during specified study period.<br><br>Exclusion: other tumours or osteosarcomas involving other locations	Not clear<br><br>Patient identified through medical record.	Interview (detailed history of symptom onset)<br><br>Symptoms before presentation	Full text article and raw data	Raw data including individual patient symptoms.<br><br>Extensive symptom list (7)
Parkes et al[16]	All malignancy diagnosed in bone<br><br>Inclusion/exclusion criteria: Yes	The West Midlands Regional Children's Tumour Registry	Yes.<br><br>Pathology review where material was available	Cancer registry data + Medical notes reviewed by clinicians<br><br>Pre-diagnostic/ clinical presentation at diagnosis	Full text article	Extensive symptoms list (7)
Guillon et al[17]	Osteosarcoma<br><br>Inclusion/exclusion criteria: Yes	All consecutive patients during specified study period from 7 tertiary centres.	Yes.<br><br>Histologically confirmed	Medical record (secondary/ tertiary)<br><br>Pre-diagnostic/ clinical presentation at diagnosis	Full text article	Symptoms list (3)
Akhavan et al[18]	Ewing's/ PNET<br><br>Inclusion/exclusion criteria: Yes	All consecutive patients during specified study period.	Yes.<br><br>Documented pathology report	Medical record (hospital setting not specified)<br><br>Symptoms recorded; timing not specified	Full text article	Symptoms list (3)
Brasme et al[19]	Ewing's/ PNET<br><br>Inclusion/exclusion criteria: Yes	All patients from two prospective multi-centre trials (EW88 and EW93)	Histologically confirmed	Multi-centre trial data<br><br>Pre-diagnostic/ clinical presentation at diagnosis	Full text article	Symptoms list (5)
Berlanga et al[20]	High grade osteosarcoma<br><br>Inclusion/exclusion criteria: Yes	All consecutive patients during specified study period	Yes.<br><br>Histologically confirmed	Medical record (secondary/ tertiary)<br><br>Pre-diagnostic/ clinical presentation at diagnosis	Full text article	Symptoms list (3)

Authors	Study population	Sample strategy	Case definition and verification	Ascertainment of data	Type of report	Level of symptom detail reported
Misra et al [21]	Ewing's	All consecutive patients during specified study period	Not reported	Medical record (secondary/ tertiary)<br><br>Pre-diagnostic/ clinical presentation at diagnosis	Conference abstract	Extensive symptoms list (7)
Caloretti et al[22]	Ewing's	All consecutive patients during specified study period	Not reported	Not specified	Conference abstract	Symptoms list (4)
Nazeer et al. [23]	All Ewing sarcoma	All consecutive patients during specified study period from 2 tertiary centres.	Yes.<br><br>Histologically confirmed	Medical record (secondary/ tertiary)<br><br>Pre-diagnostic/ clinical presentation at diagnosis	Full text article	Limited symptoms list (2)
Galila et al. [24]	Osteosarcoma and Ewing's<br>Inclusion/exclusion criteria: Yes	All consecutive patients during specified study period	Yes<br><br>Histologically confirmed	Medical record and Interview (secondary/ tertiary)<br><br>Pre-diagnostic/ clinical presentation at diagnosis	Full text article	Extensive symptoms list (13)
Geiszl et al[25]	Ewing's<br><br>Inclusion/exclusion criteria: Yes	All consecutive patients during specified study period.<br><br>The centre treated >90% of the cases in Hungary during the study period	Yes<br><br>Diagnosis extracted from medical records; details unspecified.	Medical record (secondary/ tertiary)<br><br>Pre-diagnostic/clinical presentation at diagnosis	Full text article	Symptoms list (5)
Chen et al [26]	Intracranial Ewing sarcoma/peripheral PNET<br><br>Inclusion/exclusion criteria: Yes	All consecutive patients during specified study period	Yes<br>Histologically and molecular diagnosis	Medical record (secondary/ tertiary)<br><br>Symptoms recorded; timing not specified	Full text article	Symptoms list (4)
Majeed et al[27]	Ewing sarcoma<br><br>Inclusion/exclusion criteria: Yes	All consecutive patients during specified study period	Yes<br>Histopathologic diagnosis	Medical record (hospital setting not specified)<br><br>Pre-diagnostic/clinical presentation at diagnosis	Full text article	Symptoms list (3)
Liveralla et al [28]	Extraosseous Ewing's<br><br>Inclusion/exclusion criteria: Yes	All consecutive patients during specified study period	Yes.<br>Histological and molecular diagnosis	Medical record (secondary/ tertiary)<br><br>Pre-diagnostic/clinical presentation at diagnosis	Full text article	Limited symptoms list (2)
