# Supplementary material for: Clinical presentation of bone tumours in children and young people: a systematic review and meta-analysis
Source: Arch Dis Child. 2025 Feb 5;110(8):e327879. doi: 10.1136/archdischild-2024-327879 (PMC12320600; doi:10.1136/archdischild-2024-327879)
Supplement: online supplemental file 5 [file archdischild-110-8-s005.pdf]

Table S5 Pooled proportions and 95% confidence interval of all signs and symptoms

Symptom	n	Pooled % (95% CI)	*Heterogeneity (I^2)*
Pain	1091	64 (52-75)	95
Swelling	303	22 (6-42)	98
Fever	111	3 (0-8)	92
Pain and swelling	66	3 (0-8)	95
History of trauma	75	2 (0-7)	93
Pathology fracture	45	2 (0-5)	81
Palpable mass	282	2 (0-11)	98
Functional limitation	37	2 (0-5)	92
Volume increase	142	2 (0-8)	97
Limp	32	2 (0-4)	87
Mass	15	1 (0-3)	76
Weight loss	40	1 (0-3)	85
Nerve compression	73	1 (0-4)	91
Pain on weight bearing	13	1 (0-3)	77
Painless swelling	13	1 (0-2)	69
Lymph node involvement	14	1 (0-2)	73
Cauda equina/spinal cord compression	29	1 (0-2)	73
Pain at rest	19	1 (0-2)	70
Respiratory distress	9	1 (0-2)	65
Headache with vomiting	7	1 (0-2)	61
Neurological manifestation	7	1 (0-1)	49
Pain worse at night	7	1 (0-1)	53
Inflammation	6	0.5 (0-1)	48
Inability to walk	5	0.4 (0-1)	31
Systemic manifestation	5	0.4 (0-1)	31
Cough	4	0.3 (0-1)	22
Sphincter dysfunction	4	0.3 (0-1)	16
Headache	2	0.2 (0-1)	0
Paraesthesia	2	0.2 (0-1)	0
Limited mouth opening	2	0.2 (0-1)	0
Fast growing growth	2	0.2 (0-1)	0
Horner's syndrome	2	0.2 (0-1)	0
Claudication	2	0.2 (0-1)	0
Loss of appetite	1	0.2 (0-1)	0
Eye proptosis	1	0.2 (0-1)	0
Dizziness and weakness	1	0.2 (0-0)	0
